# Developing an App to Screen for Dual Disorders: A Tool for Improving Treatment Services in Mexico

**DOI:** 10.3389/fpsyt.2021.697598

**Published:** 2021-10-29

**Authors:** Rodrigo Marín-Navarrete, Marta Torrens, Aldebarán Toledo-Fernández, Joan Ignasi Mestre-Pinto, Ricardo Sánchez-Domínguez, Alejandro Pérez-Lopez, Ricardo Saracco-Alvarez, Eduardo Ángel Madrigal-De León, Nestor Szerman

**Affiliations:** ^1^Unidad de Ensayos Clínicos en Adicciones y Salud Mental, Instituto Nacional de Psiquiatría Ramón de la Fuente Muñiz, Mexico City, Mexico; ^2^Programa de Investigación en Neurociencias, Institut Hospital del Mar d'Investigacions Mèdiques, Barcelona, Spain; ^3^Centro Anáhuac de Investigación en Psicología, Facultad de Psicología, Universidad Anahuac Mexico, Mexico, Mexico; ^4^Hospital Gregorio Marañón, Servicio de Salud Mental Retiro, Madrid, Spain

**Keywords:** dual disorders, substance use disorders, treatment, screening, m-Health, ICTs

## Abstract

**Background:** Previous studies in Mexico undertaken at residential facilities for treating substance use disorders (SUDs) reported that the prevalence of Dual Disorders (DDs) is over 65%. DDs pose a major challenge for the Mexican health system, particularly for community-based residential care facilities for SUDs, due to the shortage of certified professionals to diagnose and treat these patients. Moreover, the lack of standardized algorithms for screening for and evaluating DDs to refer patients to specialized services (whether private or public) hinders timely care, delaying the start of integrated treatment. The use of new technologies provides a strategic opportunity for the timely detection of DDs through the development of standardized digital applications for the timely detection of DDs.

**Objective:** To develop an app to screen for DDs, which will contribute to referral to specialized services in keeping with the level of severity of psychiatric and addictive symptomatology, and be suitable for use by community-based residential care facilities for SUDs.

**Method:** The research project was implemented in two stages. Stage 1 involved obtaining the psychometric properties of the Dual Diagnosis Screening Interview (DDSI). Stage 2 consisted of two steps to test the *Beta* version of the app and the quality of version 1.0.

**Results:** The DDS obtained sensitivity and specificity scores above 85%. The app and its algorithm to screen for and refer DDs proved to be efficient and easy to apply with satisfactory community acceptance.

**Conclusion:** The app promises to be a useful screening tool at residential addiction treatment centers.

## Background

### Overview of Dual Disorders

Several clinical research studies have reported high prevalence of Dual Disorders (DDs) or Co-occurring Disorders (the clinical correlation between substance use disorders and other psychiatric disorders) (1–3). Patients with DDs have complex symptomatology that produces a synergistic effect between the two dimensions, increasing the level of severity and negatively impacting those who suffer them (4). DDs are associated with a higher risk of sexually transmitted infections, suicidality (ideation, behavior and attempts), school and work dropout, legal problems, and greater biopsychosocial deterioration (5).

Despite the reports, there are barriers to accessing adequate treatment. The scientific literature has identified two broad categories to service access: personal characteristics and structural barriers. Personal characteristics include personal vulnerabilities associated with the symptomatology and severity of both psychopathological dimensions, in addition to the motivation to change and personal beliefs associated with preconceived ideas about health professionals, stigma and cultural differences (6). The review identified the following structural barriers: service availability, DD identification, service provision, racial and ethnic disparities and last but not least, insurance policies (6).

All treatment programs must offer standardized screening and assessment services to determine the locus of attention or level of care required by the needs and particularities of each patient (3).

### Screening, Assessment, and Treatment Planning for People With DDs

Screening, assessment, and treatment planning constitute an interrelated process designed to explore, detect, inform, refer, diagnose, and plan the treatment of people with DDs in the best program available according to the needs of each patient. Understanding these three components (screening, assessment, and treatment planning) as an integrated process is key to successful treatment (7).

The screening component is a formal process that determines the likelihood that a patient with SUD will present signs, symptoms, or behaviors associated with other mental disorders or vice versa. Its purpose is not to establish a specific diagnosis, but to recognize the need for an in-depth assessment. The assessment component determines differential diagnoses and identifies other clinical characteristics such as readiness for change, strengths or problematic areas that may affect treatment and rehabilitation, and engages the patient in the treatment. Finally, the treatment planning component integrates treatment programs and interventions for both dimensions. The plan is tailored to the individual needs, readiness to change, preferences, and personal goals of the patient (7).

Despite evidence-based recommendations, not all programs have the same screening, assessment, and treatment services. Likewise, the range of competencies and expertise among health professionals varies enormously. Both aspects are obstacles to undertaking an integrative process. It is therefore essential to implement standardized procedures and tools to screen for and assess DDs according to the *locus* of care of each program (3).

### Digital Tools for Screening Mental Disorders

The advantages of using screening tools include their ease of use and scoring, the limited training required for their administration, and, for well-researched tools, a known level of reliability and available cut-off scores. One disadvantage of screening instruments is that they sometimes become the only component of the screening process. A second disadvantage is that a routinely administered screening instrument provides little opportunity to establish a connection with the patient. It is important to encourage patients to accept a referral for assessment and treatment if needed (7).

The growth of information and communication technologies in the past decade has spawned a revolution in the detection, assessment and treatment procedures of various medical specialties by promoting the development of multiplatform telehealth (telemedicine) methodologies and tools (e-health), as well as mobile health (m-health) featuring the use of mobile devices (such as smartphones, tablets, laptops and monitoring devices) due to their ease of access, portability, functionality and usability (8, 9).

In the field of mental health, apps have been developed for a range of purposes. Some offer self-directed treatment services and live video conferences for psychoeducation for patients and families, psychiatric and/or psychological treatment (counseling or psychotherapy) in individual or group modalities and self-help groups (10). There are also apps designed to screen for and/or evaluate mental health problems and addictions. These applications can be incorporated into standardized self-report instruments (scales and questionnaires) and/or used with the help of a health professional (structured or semi-structured interviews) (8, 11).

Screening apps generally use standardized instruments to screen for and monitor psychiatric symptoms (such as depression, anxiety, mania, psychosis, impulsivity and self-harm) and substance use (including aspects such as frequency, quantity, severity and craving) (12–15).

There is evidence, however, that self-report/self-monitoring apps for screening are inaccurate in that they fail to provide cut-off points, or use non-standardized instruments. (14, 16). Likewise, they may offer inappropriate recommendations for the real needs of users (potential patients), by over- or under-estimating the actual state of health of the user (10). Moreover, it has been reported that about half the applications found in stores (App Store and Google Play) make claims about their effectiveness, although the evidence they present is based mainly on bibliographic searches with little methodological rigor or on personal involvement in their development (14).

Despite significant progress, the range of apps for DD screening is limited. To our knowledge, the only app for this purpose is the DDSI (Dual Diagnosis Screening Interview). This application is a practical tool for clinicians and based on a structured interview to screen for the following disorders: generalized anxiety, panic attack, agoraphobia, social phobia, specific phobias, posttraumatic stress disorder (PTSD), major depression, persistent depression (dysthymia), mania, psychosis, and attention deficit/hyperactivity disorder (ADHD) (17), but 2 years ago it stopped being available to download from commercial stores.

### Dual Disorders in Mexico

The Mexican public network for the treatment of SUDs and their attendant problems comprises over 400 outpatient and 30 hospitalization units (2). However, as in other Latin American countries, state capacity for dealing with health problems is limited (18), particularly in patients with high levels of symptom severity.

For this reason, in Mexico, about a third of those with SUDs receive treatment at community-based residential care facilities for SUDs. These facilities (totaling over 2,000) are a popular alternative for recovery from addictions since many of them are designed to meet service demand quickly, particularly among patients in a state of substance intoxication with agitated or disruptive behavior. They are located in marginal areas, which makes them more affordable. Their services are much cheaper than private professional services, which most of those affected are unable to afford. They are usually based on the 12-step model of Alcoholics Anonymous (peer-to-peer care), offering treatment of varying lengths ranging from 3 to 12 months (19, 20).

Official reports indicate that about 91% of subjects receiving treatment in these settings are men aged between 18 and 30. Five per cent have used injection drugs, 64% report having shared needles despite being aware of the health risks and at least 2% are living with HIV (21). Other studies have found that about 75% of individuals have shown a lifetime prevalence of DDs (22), while over 60% have done so in the past 30 days (2).

However, despite their strengths, the majority of these community centers face significant constraints due to their limited physical and technological infrastructure, insufficient funds, untrained staff, and lack of standardized screening, assessment and treatment planning procedures and mental health professionals (19, 20).

The scientific literature has widely documented the potential problems faced by non-specialized health personnel when evaluating and treating people with mental health problems and addictions, especially when they lack training and supervision (23–25). In this respect, we inferred that the peer-to-peer care model with untrained staff and a lack of standardized procedures for treating patients with DDs increases the risk of iatrogenic harm, malpractice and negligence (26–28).

Nonetheless, despite their structural limitations and lack of specialized personnel, community-based residential care facilities for SUDs are in high demand for those with DDs. As a result, the Mexican government has made great efforts to regulate services by establishing minimum operating standards (29), and to certify the skills of paraprofessional staff (peers) (30). According to the evidence, it is essential to implement screening services as part of a standardized admissions process. The foregoing is intended to prevent, as far as possible, the presence of malpractice and negligence, and to reduce the risk of iatrogenic harm due to the lack of skills of paraprofessional staff (peers) and the shortage of qualified health personnel to assess and treat patients with DDs.

The aim of this study is to develop an app to screen for DDs that will improve the services offered by community-based residential care facilities for SUDs in Mexico, using an algorithm to detect the degree of severity of psychiatric and addictive symptomatology, and to contribute to the detection, counseling and referral of patients to specialized services.

## Methods

The research project was implemented in two stages. The purpose of Stage 1 was to obtain the psychometric properties of the Dual Diagnosis Screening Interview (DDSI), and to determine its validity by comparing it to a gold standard such as the Mini International Neuropsychiatric Interview (MINI). Stage 2 consisted of two steps to test the *Beta* version of the app and the quality of version 1.0.

All the study procedures, informed consent, evaluation forms and recruitment materials used were approved by the Ethics and Research Committee of the Ramón de la Fuente Muñiz National Institute of Psychiatry (INPRFM) (No. IC17055.0) and adhered to the recommendations of the World Medical Association, and the Declaration of Helsinki on international good practices for research in human beings.

### Stage 1

#### Design, Sites, and Participants

A cross-sectional study with a convenience sample was implemented at 33 community-based residential SUD treatment facilities in Mexico City. Eligible participants met the following criteria: men and women aged ≥18 years, who had spent at least seven days in residential treatment to control for the residual effects of recent intoxication, who were literate and had signed the informed consent form. Participants with disabling symptoms of psychosis, mania (MINI) (31) and cognitive impairment (MoCA) (32) were excluded. Data were collected between February and December 2017.

#### Measures

##### Sociodemographic Data and Substance Use

This questionnaire is based on the Addiction Severity Index (33), following the recommendations of Mäkelä (34). The sections included sociodemographic data (age, sex, education, marital status, and source of economic income), substance use (impact substance, age of onset, years of use, use in the past 30 days, route of administration, and days of abstinence) and health service use (type of health service, health issue, and professional help).

##### Mini International Neuropsychiatric Interview (MINI)

This structured interview is used for the rapid, accurate diagnosis of psychiatric disorders according to DSM-IV criteria (31). An adapted, Spanish-language version was used. For this study, the following lifetime diagnoses from the MINI version were used in the analyses: mania/hypomania episode, psychotic disorder, depressive disorder (major episode or dysthymia), suicide attempt, alcohol use disorder, drug use disorder, PTSD, anxiety disorders (panic disorder, general anxiety disorder, specific or social phobia), antisocial personality disorder, and adult ADHD.

##### Dual Diagnosis Screening Interview (DDSI)

This brief interview evaluates the most frequent and severe psychiatric disorders found in substance users: depression, dysthymia, mania, psychosis, panic disorder, agoraphobia, simple phobia, social phobia, generalized anxiety disorder, PTSD, and ADHD, and lasts 13–20 min. The DDSI has shown a sensitivity of >80%, and a specificity of >82% for the identification of lifetime disorders such as depression, mania, psychosis, social phobia and specific phobia (17).

#### Procedures

##### Site Selection

The 33 selected facilities all complied with current Mexican regulations for addiction treatment (NOM-028-SSA2-2009), were willing to facilitate the study procedures, and were equipped with adequate facilities to ensure patient privacy during study assessments.

##### Field Team, Training, and Certification

The field team comprised psychologists, six interviewers with undergraduate studies, and two supervisors with graduate studies in clinical psychology. All members of the clinical team had been trained and certified in the procedures and evaluation of the study. Training consisted of a centralized five-day program, comprising theoretical and practical seminars. Certification was undertaken through a role-play exercise. Training and certification were provided by the research team.

##### Recruitment and Enrollment of Participants

Potential participants were recruited through a group discussion in which they were informed of the characteristics of the study. Interested participants underwent an individual informed consent and signing process, with an interviewer providing detailed information on the study, its risks and benefits, and subjects' rights. If they agreed to participate, subjects proceeded to sign the informed consent form and the interview began to be administered (MINI and DDSI), which took ~2 hours. The interval between the administration of both instruments ranged from 3 to 5 days.

#### Statistical Analysis

The groups with and without DD were formed based on the presence of a psychiatric disorder identified by the DDSI. Univariate analyses were performed for demographic variables (sex, age, education, marital status) and substance use (in the past 30 days), chi-square (χ^2^) for categorical variables and Student's *t* for numerical variables. DDSI efficiency (sensitivity, specificity, positive and negative predictive values) of the predictive variable in relation to the MINI was calculated and tested for statistical significance using a 2 × 2 Chi-Square test. Since missing data were assumed to be completely random (MCAR), we used multiple imputation for the missing data with the R statistical package. A significant value of *p* < 0.05 was used. All statistical analyses were performed using SPSS v23.

### Stage 2

Stage 2, comprising two steps, was implemented between January 2018 and December 2019, to test and improve the components of the *Beta* version and test the quality of the 1.0 version of the app.

#### Step 1

After the psychometric properties of the DDSI had been obtained, the *Beta* version of the App was developed, which comprised basic functions and components such as patient file control, personal data, and substance use pattern forms, DDSI, and results reports.

To test the functionality and usability of the *Beta* version, the field team administered just over 60 interviews to identify possible errors and improvements. SCRUM (35) methodology was used to improve the interaction between the teams [field, research and Information and Communication Technologies (ICT)]. Accordingly, the necessary minimum was documented through user stories, and user acceptance tests (UAT) were much more dynamic, with rapid feedback for error correction.

To identify possible improvements to the procedures and components of the *Beta* version, working meetings were held between teams (field, research, and ICT). Results were collected at the end of the *Beta* version test, and it was deemed fit to generate usability reports and errors. Taking this as process input, activities were classified as error debugging, usability improvement, and component modification or the addition of new components.

Through the usability testimonials of the field, research, and ICT teams, needs for improvement were grouped into four categories: ability to adapt (enables the user to understand whether the software is suitable for their needs); learning capacity (enables the user to learn how to use it quickly and intuitively). Ability to be used (enables the user to operate and control the software with ease), and aesthetics of the user interface (is pleasing and satisfies the user's interaction with the application and visual elements are easy to locate).

Likewise, during the collaborative working sessions, the need to modify certain features and incorporate new ones was detected. To this end, clinical experts outside the project were consulted to validate and strengthen improvements. Modifications and additions of components were made according to the constraints of the project (time, personnel, and funds).

#### Step 2

To test the quality of version 1.0 of the app., the Mobile App Rating Scale (MARS) (36) was used in a group of 63 addiction and mental health professionals. This scale uses the mean scores of the engagement, functionality, aesthetics, information and subjectivity subscales, and MARS is scored according to the overall mean app quality score. MARS's psychometric properties have shown excellent internal consistency (Cronbach alpha = 0.90) with the total score and internal consistencies of the subscales also being very high (Cronbach alpha = 0.80–0.89, median 0.85) (36).

To achieve this goal, 100 addiction and mental health professionals from a state treatment agency were invited to participate by email. The invitation explained the dynamics of participation, which involved (1) participating in a video conference (ZOOM system) to explain the app and its usefulness. (2) Additionally, participants with Android devices were invited to participate in a demonstration of the installation and use of the app. (3) Android device users received an email with a file to install the app (*pd-s_v1.apk*), an installation video and a link to participate in the MARS online survey (using the SurveyMonkey system). The email also stated that participation was voluntary and anonymous, and that informed consent would be requested.

## Results

### Stage 1

A total of 213 participants (94.8 % male), with an average age of 31.09 (*sd* = 11.12) years, the majority of whom had completed junior and senior high school (87.8%), and nearly half of whom (47.6%) were single, (see Table 1) were selected. The group with DDs reported a lower age (30.20 years), had completed junior or senior high school (90%) and were mostly single (53.4%) in comparison with the group without dual disorders. In both groups, the main source of income was subordinate work followed by independent work, with the only statistically significant difference found between the groups being marital status (${\text{χ}}_{\left(2\right)}^{2}$ = *6.81, p* = *0.026*). As for substance use, the substance with the greatest impact on participants with DDs was alcohol (34.2%), followed by cocaine (32.2%) and inhalants (18.1%), whereas in the group without DDs, the substance with the greatest impact was alcohol (37.5%), followed by cocaine (34.5%). No statistically significant differences were found between the two groups for use in the past month (see Table 1).

**Table 1:** Participants' characteristics (*n* = 213).

	**With** **dual disorder** ***n*** **= 149**	**Without** **dual disorder** ***n*** **= 64**	**Total** ***n*** **= 213**	***Statistical differences***	***p***
	$\tilde{x}(sd)/n(\%)\;$	$\tilde{x}(sd)/n(\%)\;$	$\tilde{x}(sd)/n(\%)\;$		
Age	30.20 (10.99)	33.15 (11.21)	*31.09 (11.12)*	***t***_**l(211)**_ **=** 4.43^*^	***0.007***
Sex				$\chi_{\left(1\right)}^{2}$ = *2.42*	*0.146*
Men	139 (93.3)	63 (98.4)	202 (94.8)		
Women	10 (6.7)	1 (1.6)	11 (5.2)		
Educational attainment				$\chi_{\left(3\right)}^{2}$ = *7.63*	*0.070*
None	0 (0)	3 (4.7)	3 (1.4)		
Elementary school	15 (10.1)	7 (10.9)	22 (10.3)		
High school	119 (79.9)	46 (71.9)	165 (77.5)		
College	15 (10.1)	8 (12.5)	23 (10.8)		
Marital status				$\chi_{\left(2\right)}^{2}$ **=** 6.81^*^	***0.026***
Single	79 (53.4)	22 (34.4)	101 (47.6)		
Married/living together	34 (23.0)	23 (35.9)	57 (26.9)		
Widowed/Divorced/Separated	35 (23.6)	19 (29.7)	54 (25.5)		
Main source of economic income				$\chi_{\left(4\right)}^{2}$ =*4.16*	*0.112*
Employment	65 (43.6)	35 (54.7)	100 (46.9)		
Self-employment	5 (3.4)	3 (4.7)	8 (3.8)		
Family support	58 (38.9)	19 (29.7)	77 (36.2)		
Other	19 (12.8)	5 (7.8)	24 (11.3)		
None	2 (1.3)	2 (3.1)	4 (1.9)		
Impact substance				$\chi_{\left(4\right)}^{2}$ **=** 9.22^*^	***0.033***
Alcohol	51 (34.2)	24 (37.5)	75 (35.2)		
Cocaine	48 (32.2)	22 (34.5)	70 (32.9)		
Marijuana	17 (11.4)	11 (17.2)	28 (13.1)		
Inhalants	27 (18.1)	6 (9.4)	33 (15.5)		
Other	6 (4.0)	1 (1.6)	7 (3.3)		
Use in past 30 days					
Alcohol	13.29 (11.7)	10.13 (10.37)	12.36 (11.39)	*t_(198)_ = 1.79*	*0.070*
Cocaine	11.17 (12.25)	10.07 (12.61)	10.87 (12.32)	*t_(165)_ = 0.51*	*0.435*
Marijuana	13.41 (13.75)	9.74 (12.66)	12.42 (13.53)	*t_(158)_ = 0.15*	*0.732*
Inhalants	9.06 (12.15)	7.66 (11.88)	8.77 (12.06)	*t_(113)_ = 0.50*	*0.360*
Other	5.27 (10.01)	7.66 (12.15)	5.74 (10.42)	*t_(90)_ = −0.87*	*0.404*

* *p < 0.05. Bold indicates statistically significant values*.

#### Psychometric Properties of DDSI

According to the results, the most prevalent diagnoses were depression (35.2%), PTSD (22.5%) and ADHD (21.1%). Additionally, the psychometric properties yielded sensitivity scores ranging from 0.81 for PTSD and psychosis to 0.88 for GAD, mania, and social phobia. Specificity scores were 0.85 or higher for most of the disorders screened. Finally, negative predictive values were <0.93 whereas positive predictive values were <0.54 for most of the disorders screened (see Table 2).

**Table 2:** Psychometrics properties of DDSI (*n* = 213).

	**Sensitivity**	**Specificity**	**PPV**	**NPV**	**TN**	**FN**	**TP**	**FP**	**Prevalence %**
Depression	0.84 (0.75–0.92)	0.77 (0.69–0.84)	0.66 (0.56–0.76)	0.89 (0.83–0.95)	121	17	63	12	35.2
GAD	0.88 (0.74–0.91)	0.85 (0.79–0.90)	0.45 (0.30–0.59)	0.98 (0.95–0.98)	159	28	23	3	12.3
PTSD	0.81 (0.69–0.93)	0.75 (0.68–0.82)	0.48 (0.37–0.60)	0.93 (0.88–0.97)	124	41	39	9	22.5
Social Phobia	0.88 (0.69–0.94)	0.85 (0.80–0.90)	0.34 (0.19–0.50)	0.98 (0.96–0.99)	168	28	15	2	7.9
ADHD	0.86 (0.75–0.97)	0.89 (0.83–0.93)	0.67 (0.54–0.80)	0.96 (0.92–0.99)	149	19	39	6	21.1
PDD	0.87 (0.68–0.91)	0.92 (0.87–0.95)	0.46 (0.27–0.66)	0.98 (0.97–0.99)	181	16	14	2	7.6
Psychosis	0.81 (0.67–0.95)	0.86 (0.80–0.91)	0.54 (0.40–0.68)	0.95 (0.92–0.99)	151	25	30	7	17.3
Mania	0.88 (0.62–0.93)	0.93 (0.89–0.97)	0.38 (0.15–0.61)	0.99 (0.98–0.99)	189	13	8	1	4.2

*GAD, Generalized anxiety disorder; PTSD, Posttraumatic stress disorder; ADHD, Attention Deficit / Hyperactivity Disorder; PDD, Persistent depression disorder; PPV„ Positive predictive value; NPV: Negative predictive value; TN, True negative; FN, False negative; TP, True positive; FP, False positive*.

### Stage 2

#### Step 1

The working meetings between the field, research and IT teams resulted in a backlog report. This report contains a record of all the errors (bugs) and navigation issues detected, together with the need to modify existing components or incorporate new ones, which was prioritized by the field and research team.

**Figure 1:**
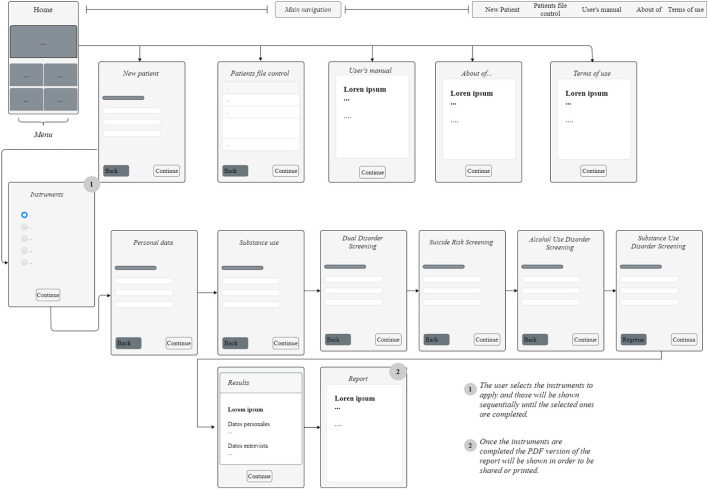
App navigation flow chart. App Name: Patología Dual Screening. Description: It is an application for mobile devices to help health professionals in the timely detection of the co-occurrence between substance use disorders with other psychiatric disorders. Version: 1.0. Update date: January / 2020. Language: Spanish. Developed by: Unit of Clinical Trials in Addictions and Mental Health of the Ramón de la Fuente Mu$\tilde{\text{n}}$iz National Institute of Psychiatry and the Institut Hospital del Mar d'Investigacions Mèdiques (IMIM) / Barcelona. Cost: The application is free to use, it only requires requesting authorization from the corresponding institutions. Hardware / software requirements: 62Mb storage space. 50 Mb in RAM memory. Android operating system version 4.4 onwards. File size: 23Mb. PDF document reader.

Some of these errors (bugs) were redundant functions, elaborate code, and runtime errors. As for usability improvement, the backlog report documented certain navigation issues between screens or regarding the arrangement of elements and the inconsistent look and feel of components, and the need to modify them or incorporate new ones (see Table 3). As a result of all the improvements implemented, the 1.0 version was created, with better performance in intuitive/ergonomic navigation (see Figure 1) than the *Beta* version.

**Table 3:** Component improvements.

**Modification of components between versions**	
***Beta*** **version**	**1.0 version**
**Patient file control**	**Patient file control**
- Record registration	- Record registration - Edit registration - Delete registration - View results report - Export Data base
**Personal data**	**Personal data**
- Sociodemographic data - Service use	- Sociodemographic data - Personal medical history - Personal psychopathology history - History of previous treatments
**Substance use pattern**	**Substance use pattern**
- Age of onset - Lifetime use - Use in past 30 days - Longer abstinence time - Administration route	- Age of onset - Lifetime use - Use in past 30 days - Longer abstinence time - Administration route
**Screening instruments**	**Screening instruments**
- Dual Disorder Screening	- Dual Disorder Screening^*^ - Suicide Screening^**^ - Alcohol Use Disorder Screening^***^ - Substance Use Disorder Screening^****^
**Results report**	**Results report**
- Visualization on mobile device	- Visualization on mobile device - Generation of results report in PDF file - Includes: Personal data, Substance use pattern, results of screenings, algorithm of recommendations, disclaimer, notes of evaluator and list of public institutions for assessment and treatment.
New component in 1.0 version	
•**Algorithm of recommendations:** Offers patients recommendations for assessment and referral to specialized treatment.	
•**User's manual:** The manual provides information on the requirements of the System, installation, navigation between screens, application interactions of the algorithm and data dictionary.	
•**Terms of use**: These provide information on the disclaimer.	
•**About:** This provides information on the research and development teams, institutions, funding, and endorsements.	
•**Data base export:** This allows the export in CSV format of the database of evaluated patients stored in the device. The database includes all the variables of the forms administered.	

Dual Diagnosis Screening Interview (DDSI) ^*^(17);

Suicide ^**^(31);

Short Alcohol Dependence Data Questionnaire (SADD) ^***^(37);

Drug Abuse Screening Test (DAST) ^****^ (38).

The most important component of the app is the traffic light algorithm designed to contribute to the rapid identification of probable psychiatric and substance use disorders, while providing the user with recommendations for assessment and referral to specialized treatment.

The algorithm assumes that screening is being carried out at a community residential treatment center where there are no specialized mental health professionals equipped to assess and treat patients with probable mental disorders other than SUDs.

**Figure 2:**
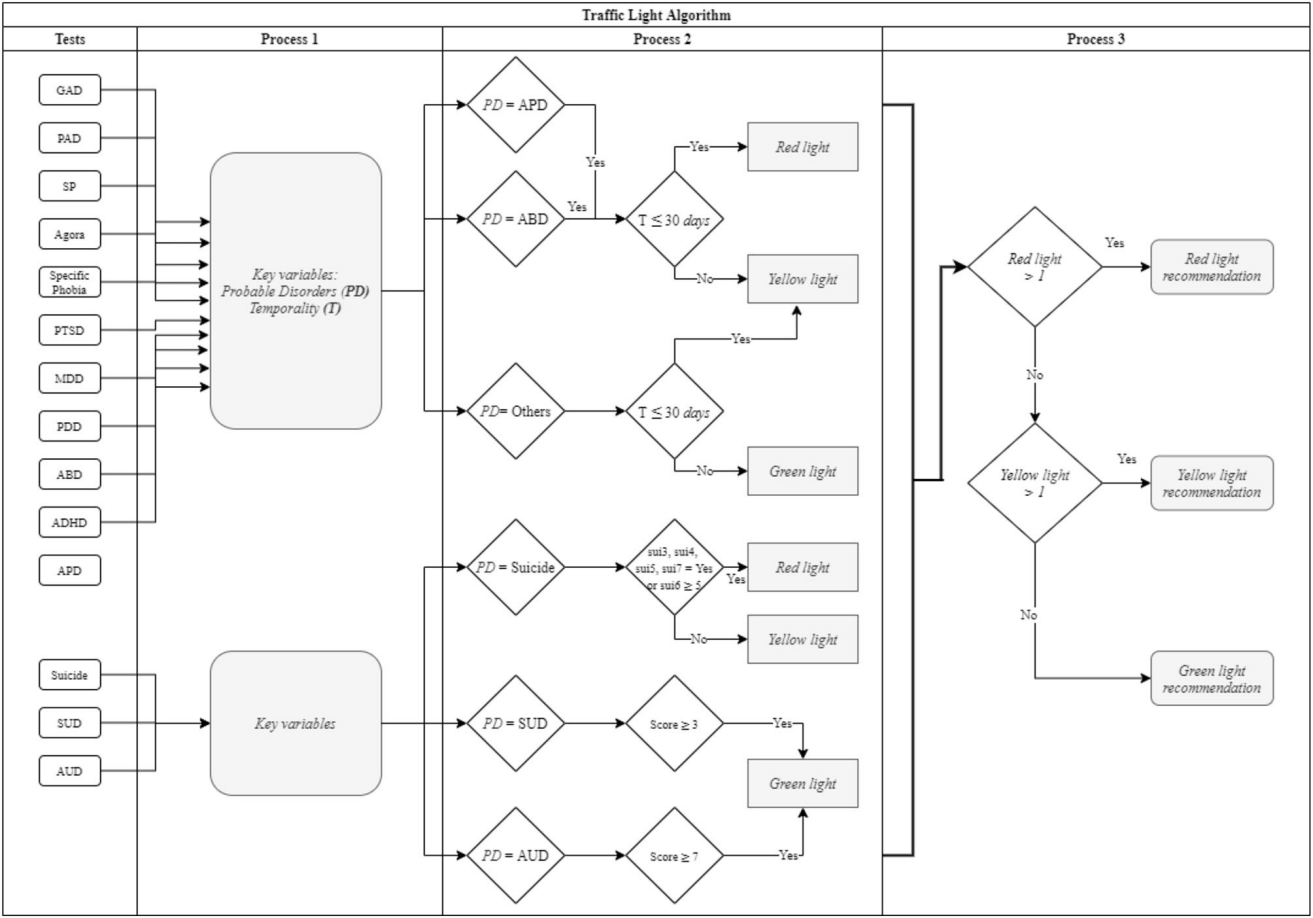
Algorithm flow chart. Traffic Light Algorithm: IF probable APD, ABD, Suicide (ideation = true OR planning = true OR attempt = true OR intentionality ≥ 5) ≤ 30 days, THEN red light recommendation ELSE IF probable APD, ABD, Suicide (ideation = true OR planning = true OR attempt = true OR intentionality ≤ 4 OR lifetime attempt) > 30 days THEN yellow light recommendation, ELSE probable APD, ABD, Suicide = false AND Others > 30 days AND SUD ≥ 3 AND AUD ≥ 7 THEN green light recommendation.

Accordingly, the purpose of the algorithm is to identify possible mental disorders and prioritize assessment and treatment needs, distinguishing between conditions that constitute a genuine psychiatric emergency (psychosis, mania and suicide) from those that do not (see Figure 2) (39).

The algorithm generates three types of recommendations, based on a traffic light system:

##### Red Light

“The patient has probable psychiatric disorders co-occurring with substance use. This condition poses a **medium to high risk**. The recommendation is to refer the patient as a priority to a specialized institution for assessment and treatment”.

##### Yellow Light

“The patient has probable psychiatric disorders co-occurring with substance use. This condition represents a **low to medium risk**. The recommendation is to refer the patient to a mental health specialist for assessment and treatment”.

##### Green Light

“The patient does not have probable mental disorders co-occurring with substance use. This condition **does not represent an apparent risk**. The recommendation is to continue with standard treatment”.

#### Step 2

A total of 100 addiction and mental health professionals were invited to participate, 85 of which agreed to participate in the videoconference and only 63 of which answered the survey. The average age of the health professionals was 39.71 years, most of whom were women. A total of 50.8% were doctors, 39.7% were psychologists and 9.5% were from other professions. Seventy-three per cent reported having completed postgraduate studies. Regarding the number of years of experience in mental health treatment, it was observed that the majority had over 6 years' experience (63.4%). Likewise, according to the MARS results, the app obtained a total quality score of 4.3. Moreover, it was observed that subjects with postgraduate studies, doctors, and those with fewer than 10 years' experience in mental health rated the app slightly lower (see Table 4).

**Table 4:** Comparisons between MARS scores and demographic variables (*n* = 63).

	**Engagement**	**Functionality**	**Aesthetics**	**Information**	**Subjectivity**	**Total**
	$\;\tilde{x}(sd)$	$\;\tilde{x}(sd)$	$\;\tilde{x}(sd)$	$\;\tilde{x}(sd)$	$\;\tilde{x}(sd)$	$\;\tilde{x}(sd)$
Education						
Undergraduate	4.3 (0.78)	4.4 (0.59)	4.1 (0.75)	4.2 (0.61)	3.6 (0.69)	4.2 (0.63)
Graduate	4.1 (0.69)	4.2 (0.54)	4.1(6.9)	4.1 (0.76)	3.4 (0.67)	4.1 (0.59)
Profession						
Psychology	4.3 (0.63)	4.5 (0.36)	4.2 (0.57)	4.3 (0.59)	3.7 (0.68)	4.3 (0.46)
Medicine	3.9 (0.78)	4.1 (0.63)	3.9 (0.82)	3.9 (0.82)	3.3 (0.69)	3.9 (0.68)
Years of experience in mental health						
0–5 years	4.1 (0.79)	4.2 (0.65)	4.1 (0.81)	4.1 (0.81)	3.3 (0.73)	4.1 (0.70)
6–10 years	4.1 (0.73)	4.1 (0.54)	3.8 (0.68)	4.0 (0.73)	3.3 (0.62)	4.0 (0.61)
+ 10 years	4.2 (0.63)	4.5 (0.34)	4.2 (0.55)	4.3 (0.61)	3.7 (0.63)	4.3 (0.44)

## Discussion

The objective of the present study was to develop an algorithm to screen for DDs, which helps detect SUDs, counsel patients and refer them to specialized services according to the level of severity of their psychiatric and addictive symptomatology, through the use of an app that will be easy to use and adopt in Community-Based Residential Care Facilities for SUDs. To this end, the specificity of the DDSI was obtained as well as its concurrent validity against the gold standard (MINI). Likewise, a *Beta* version of the App was tested, which improved version 1.0 in terms of functionality, usability, and aesthetics. It also permitted the incorporation of components to increase the detection and measurement of other psychopathological aspects, as well as making clinical decision-making more efficient by incorporating the traffic light algorithm. Finally, the MARS scale was used to enable the quality of the app to be evaluated by mental health and addiction professionals, who evaluated it positively in the five dimensions (commitment, functionality, aesthetics, information, and subjectivity).

The results obtained during Stage 1 indicate that the sensitivity and specificity scores of the DDSI were similar to those of the original study (17), where they are above 80%. Conversely, negative predictive values were higher over 90%, whereas positive predictive values ranged from 34 to 67%, These scores may be due to the fact that positive predictive values depend largely on the prevalence of a set condition (and to a lesser effect on specificity and sensitivity) as has been reported in various studies where low VPP have been obtained (40, 41). For this reason, it is estimated that when case prevalence increases, false positives fall, which makes VPPs increase. Future studies should be conducted on specific populations to determine whether VPPs increase with case numbers and do not depend on DDSI properties (42), although it may also be due to the symptoms of the addiction itself masking co-occurring psychiatric symptomatology (42). However, the DDSI can be said to be a valid, reliable screening tool suitable for use in clinical settings for the detection of possible mental disorders in people with substance use.

Additionally, results tally with those obtained in previous studies conducted in Mexico and Spain in residential and outpatient treatment centers. These data underline the fact that DD patients are the rule rather than the exception, with a prevalence of over 60% in the past 30 days. Most of them are polydrug users, with depression, anxiety, PTSD, psychotic disorders and ADHD being the most prevalent disorders (2, 17, 43, 44).

The procedure followed during step 1 resulted in version 1.0 of the App, which is significantly better than the *Beta* version, in that it eliminated recurring errors (bugs), allowing greater functionality and usability. It significantly improved the aesthetics of the interface and navigation between screens, making it more intuitive and cognitively ergonomic. In addition to the DDSI, key clinical scales (with validity, reliability, and cut-off points) were incorporated to screen for suicidality and drug and alcohol dependence (see Table 3). However, the most important feature is the inclusion of a traffic light algorithm that prioritizes the severity of the symptomatology assessed and provides recommendations according to the color of the traffic light (see Figure 1). Finally, this algorithm is linked to a detailed report of the screening process that facilitates feedback to the patient.

According to the results, it is extremely likely that the implementation of a screening service within community addiction centers in Mexico will allow non-specialized health personnel and para-professional counselors (peers) to identify, guide and, if necessary, refer patients for evaluation and treatment at a specialized public or private institution and thereby improve clinical decision-making for the benefit of patients, reducing the likelihood of negligence, malpractice and therefore iatrogenesis.

To ensure proper use of the app, institutions interested in implementing a screening service may request a 10-h in-person training package with three modules: (1) the theoretical framework of DDs, (2) basic aspects and use of the Dual Disorders Screening app and, (3) modeling and role play. This training will be available online shortly to facilitate access and achieve greater impact.

It should be noted that use of the Dual Disorders Screening app is not intended to replace professional psychiatric or psychological assessment, or the recommendations clinicians usually provide for patients. Accordingly, sensible, responsible use of the app is recommended, while communication with the treating psychiatrist should always be maintained.

### Limitations

An obvious limitation is that the Dual Disorders Screening app was only developed for Android devices. However, it is important to mention that this decision was made because the Android operating system makes it possible to install programs unavailable at virtual stores such as Google Play, in addition to the fact that Android devices are significantly cheaper than those operating with iOS.

Although the Dual Disorders Screening app is designed to be a practical tool to be used and adopted by community residential centers for addiction care in Mexico, it is understood that the app alone does not suffice. Accordingly, the development of a more robust electronic platform offering a broader range of services and products could serve as an extremely useful complement.

This platform should have e-health services such as electronic medical records compatible with RIS/PACS and LIS applications. It should also have telemedicine services for the distance training of community physicians and psychologists in the treatment of patients with DDs, as well as programs incorporating case supervision by psychiatrists and psychologists specializing in DDs. Finally, there should be m-health-based support tools for patients and community professionals.

## Conclusion

The Dual Disorders Screening app is a reliable tool for the detection of DDs, as well as the measurement of other clinical aspects associated with this population. It is hoped that the incorporation of the traffic light algorithm will enhance the clinical decision-making of personnel at community residential centers for addiction care throughout the country, thereby contributing to the improvement of public policy on addiction treatment in Mexico.

## Data Availability Statement

The original contributions presented in the study are included in the article/Supplementary Material, further inquiries can be directed to the corresponding author.

## Ethics Statement

The studies involving human participants were reviewed and approved by Ethics and Research Committee of the Ramón de la Fuente Muñiz National Institute of Psychiatry (INPRFM) (No. IC17055.0). The patients/participants provided their written informed consent to participate in this study.

## Author Contributions

RM-N and MT were the principal investigators, they designed, developed and implemented of the research project, and conduct the manuscript. AT-F and JM-P were the implementation coordinators. RS-D and AP-L were supervisors during the field work and contribute for the statistical analysis. EM-DL, RS-A, and NS made significative contributions as an experts in Dual Disorders for the elaboration of the manuscript. All authors contributed to the article and approved the submitted version.

## Funding

In his role as principal investigator, RM-N was awarded (grant number: IAPA/C-32/2016) to implement this research project by the Institute for the Care and Prevention of Addictions in Mexico City *(Spanish acronym IAPA)*. The sponsor had no role in the study design, collection, analysis, or interpretation of the data, writing the manuscript, or the decision to submit this paper for publication. During the implementation of the project and the development of this manuscript, AT-F obtained a (doctoral grant #414858), RS-D was awarded a (doctoral grant #473227) and AP-L received a (doctoral grant #449063) from the National Council of Science and Technology *(Spanish acronym CONACYT)*.

## Conflict of Interest

The authors declare that the research was conducted in the absence of any commercial or financial relationships that could be construed as a potential conflict of interest.

## Publisher's Note

All claims expressed in this article are solely those of the authors and do not necessarily represent those of their affiliated organizations, or those of the publisher, the editors and the reviewers. Any product that may be evaluated in this article, or claim that may be made by its manufacturer, is not guaranteed or endorsed by the publisher.
